# Supplementary material for: CD82 expression marks the endothelium to hematopoietic transition at the onset of blood specification in human
Source: iScience. 2023 Aug 9;26(9):107583. doi: 10.1016/j.isci.2023.107583 (PMC10484973; doi:10.1016/j.isci.2023.107583)
Supplement: Document S1. Figures S1–S9 and Table S1 [file mmc1.pdf]

iScience, Volume *26*

Supplemental information

CD82 expression marks the endothelium to hematopoietic transition at the onset of blood specification in human

Sara Menegatti, Bethany Potts, Roberto Paredes, Eva Garcia-Alegria, Syed Murtuza Baker, and Valerie Kouskoff

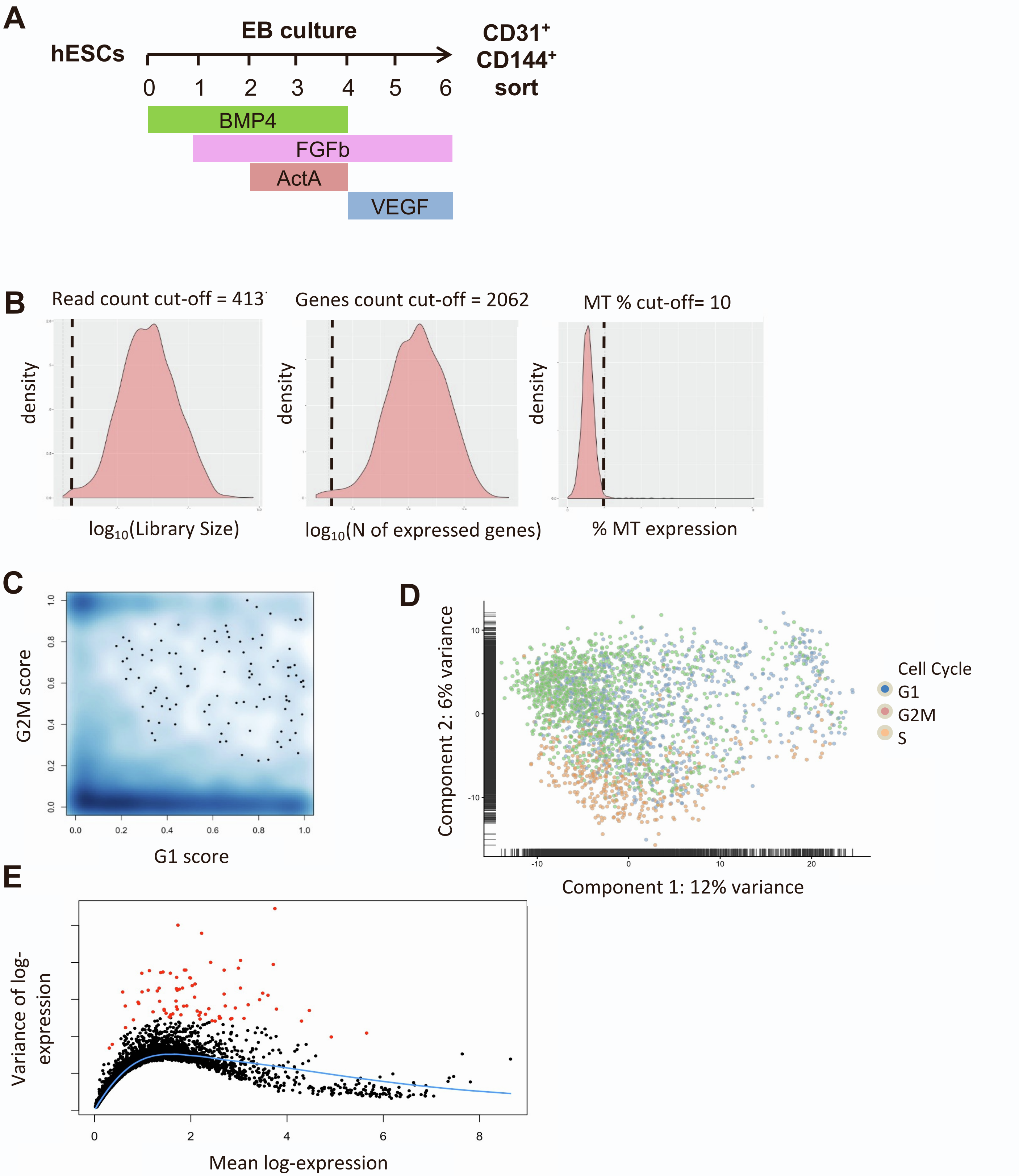
A
hESCs
EB culture
0 1 2 3 4 5 6
CD31+ CD144+ sort
BMP4
FGFb
ActA
VEGF
B
Read count cut-off = 413
Genes count cut-off = 2062
MT % cut-off= 10
density
log10(Library Size)
log10(N of expressed genes)
% MT expression
C
G2M score
G1 score
D
Component 2: 6% variance
Component 1: 12% variance
Cell Cycle
G1
G2M
S
E
Variance of log-expression
Mean log-expression

Figure S1

Figure S1. Differentiation scheme and scRNA-seq quality control. Related to Figure 1

(A) Scheme of hESC differentiation toward endothelial and haematopoietic progenitors; hESCs are cultured as 3D embryoid bodies for 6 days, with the step-wise addition of growth factors. At day 0, EB media is supplemented with 10ng/ml BMP4. At day1, 5ng/ml FGFb is added to the culture. At day2, EB media is refreshed and supplemented with BMP4, FGFb and 0.9ng/ml Activin A. At day 4, medium is refreshed and supplemented with FGFb and 12ng/ml VEGF. (B) Normal distribution of cell density for library size, number of expressed genes and mitochondrial gene expression. Dotted lines define cut-offs used to filter low-quality cells. (C) Cell cycle analysis on generated dataset. Dark areas represent high cell density. (D) PCA on generated dataset showing cell cycle classification for each cell. (E) Identification of Highly Variable Genes (HVGs, red dots).

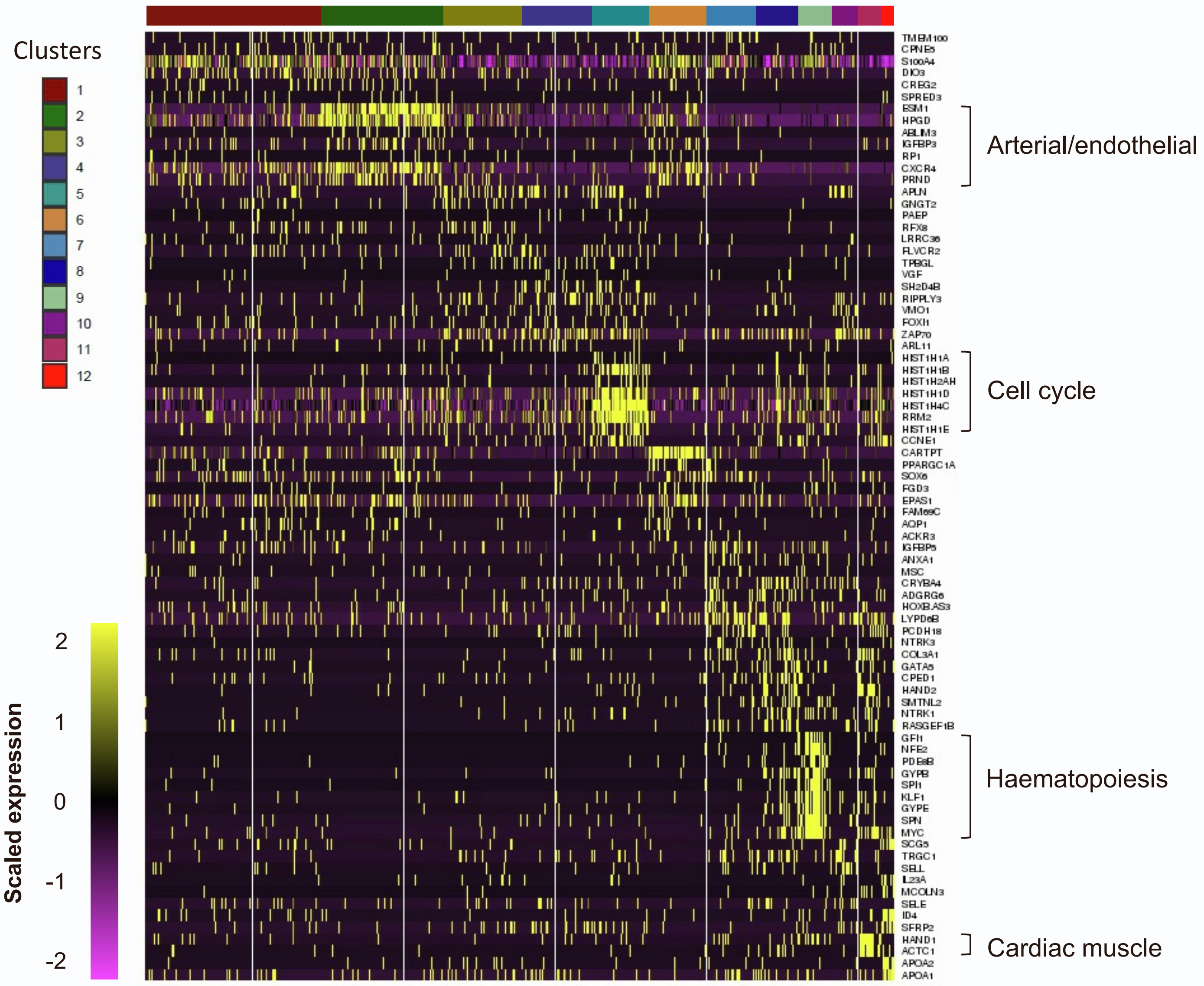

Figure S2: Heatmap of the 10 most upregulated genes in each cluster. Related to Figure 2
Grouping of arterial, cell cycle, haematopoiesis and cardiac muscle development are identifying some of the clusters included in the dataset.

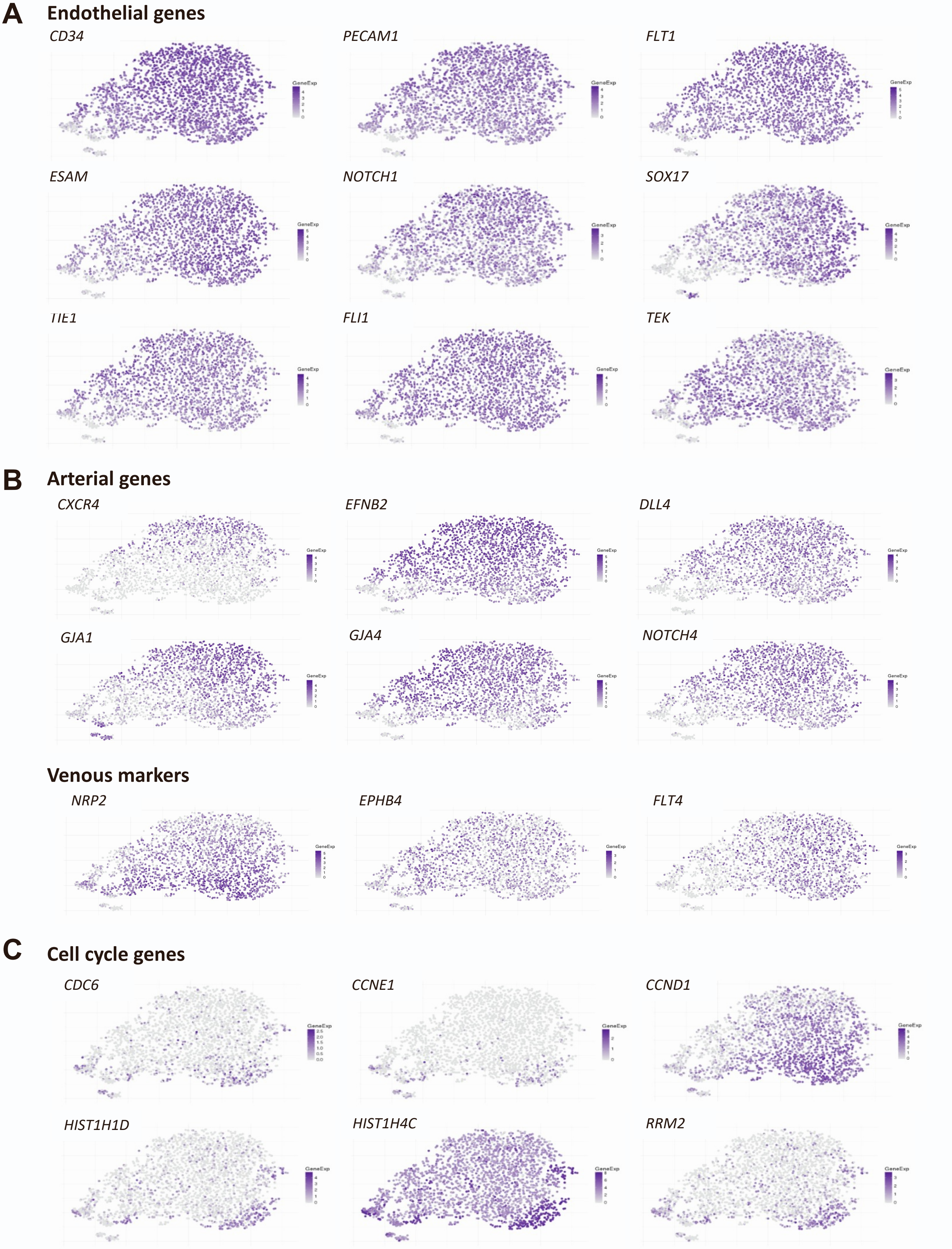
A
Endothelial genes
CD34
PECAM1
FLT1
ESAM
NOTCH1
SOX17
TIE1
FLI1
TEK
B
Arterial genes
CXCR4
EFNB2
DLL4
GJA1
GJA4
NOTCH4
Venous markers
NRP2
EPHB4
FLT4
C
Cell cycle genes
CDC6
CCNE1
CCND1
HIST1H1D
HIST1H4C
RRM2

Figure S3

Figure S3. Gene expression across clusters. Related to Figure 2.

t-SNE plots showing the expression of endothelial genes (A), arterial and venous genes (B) and cell cycles genes (C) across all clusters.

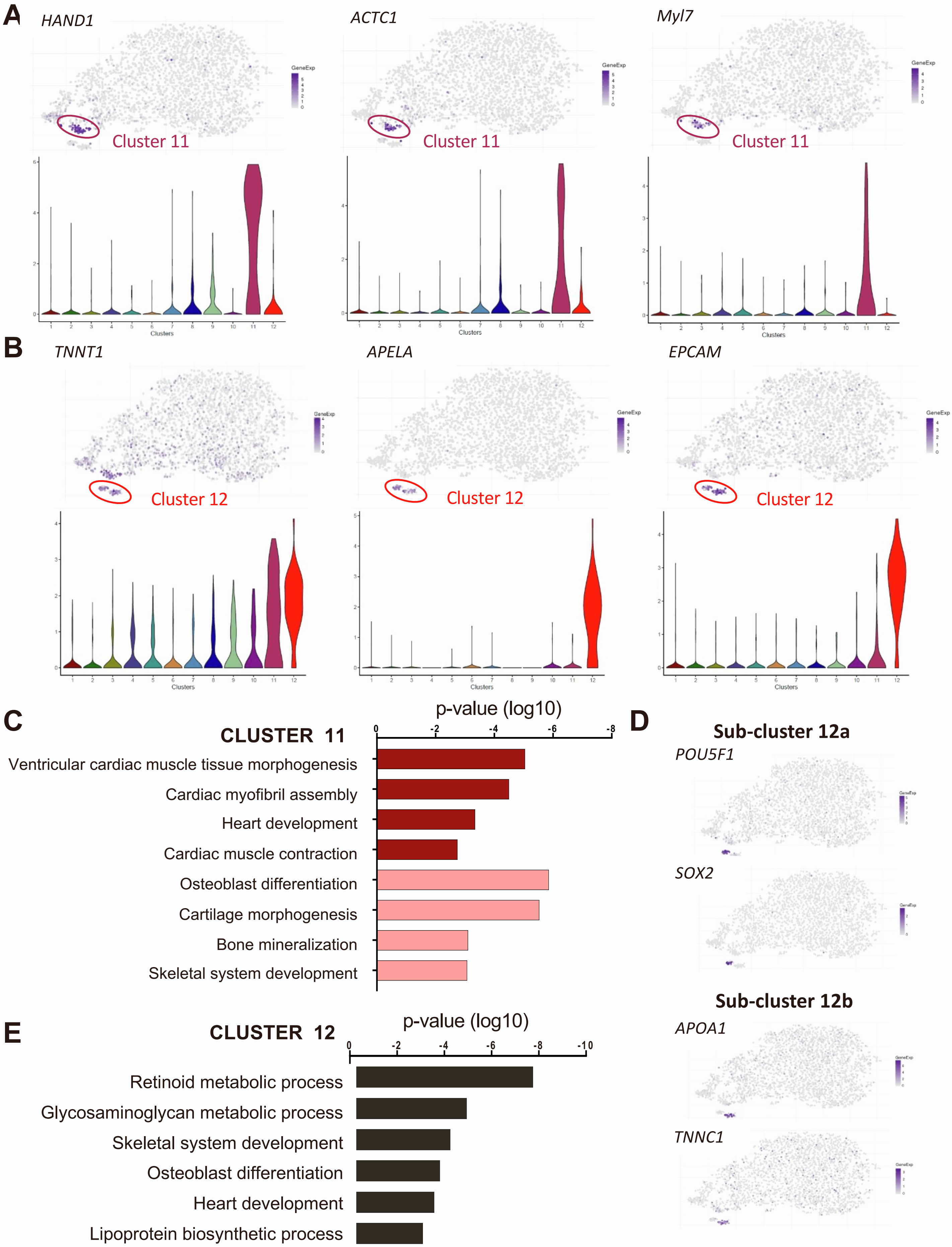
A
HAND1
Cluster 11
ACTC1
Cluster 11
Myl7
Cluster 11
B
TNNT1
Cluster 12
APELA
Cluster 12
EPCAM
Cluster 12
C
CLUSTER 11
p-value (log10)
0
-2
-4
-6
-8
Ventricular cardiac muscle tissue morphogenesis
Cardiac myofibril assembly
Heart development
Cardiac muscle contraction
Osteoblast differentiation
Cartilage morphogenesis
Bone mineralization
Skeletal system development
D
Sub-cluster 12a
POU5F1
SOX2
Sub-cluster 12b
APOA1
TNNC1
E
CLUSTER 12
p-value (log10)
0
-2
-4
-6
-8
-10
Retinoid metabolic process
Glycosaminoglycan metabolic process
Skeletal system development
Osteoblast differentiation
Heart development
Lipoprotein biosynthetic process

Figure S4

Figure S4. Characterisation of cluster 11 and 12. Related to Figure 3.

(A) t-SNE and violin plots showing the expression of *HAND1*, *ACTC1* and *MYL7* in all clusters. (B) t-SNE and violin plots showing the expression of *TNNT1*, *APELA* and *EPCAM* expression in all clusters. (C) GO terms enriched in cluster 11 upregulated genes. (D) t-SNE plots showing the expression of *POUF1*, SOX2, *APOA1* and *TNNC1* expression in all clusters. (E) GO terms enriched in cluster 12 upregulated genes.

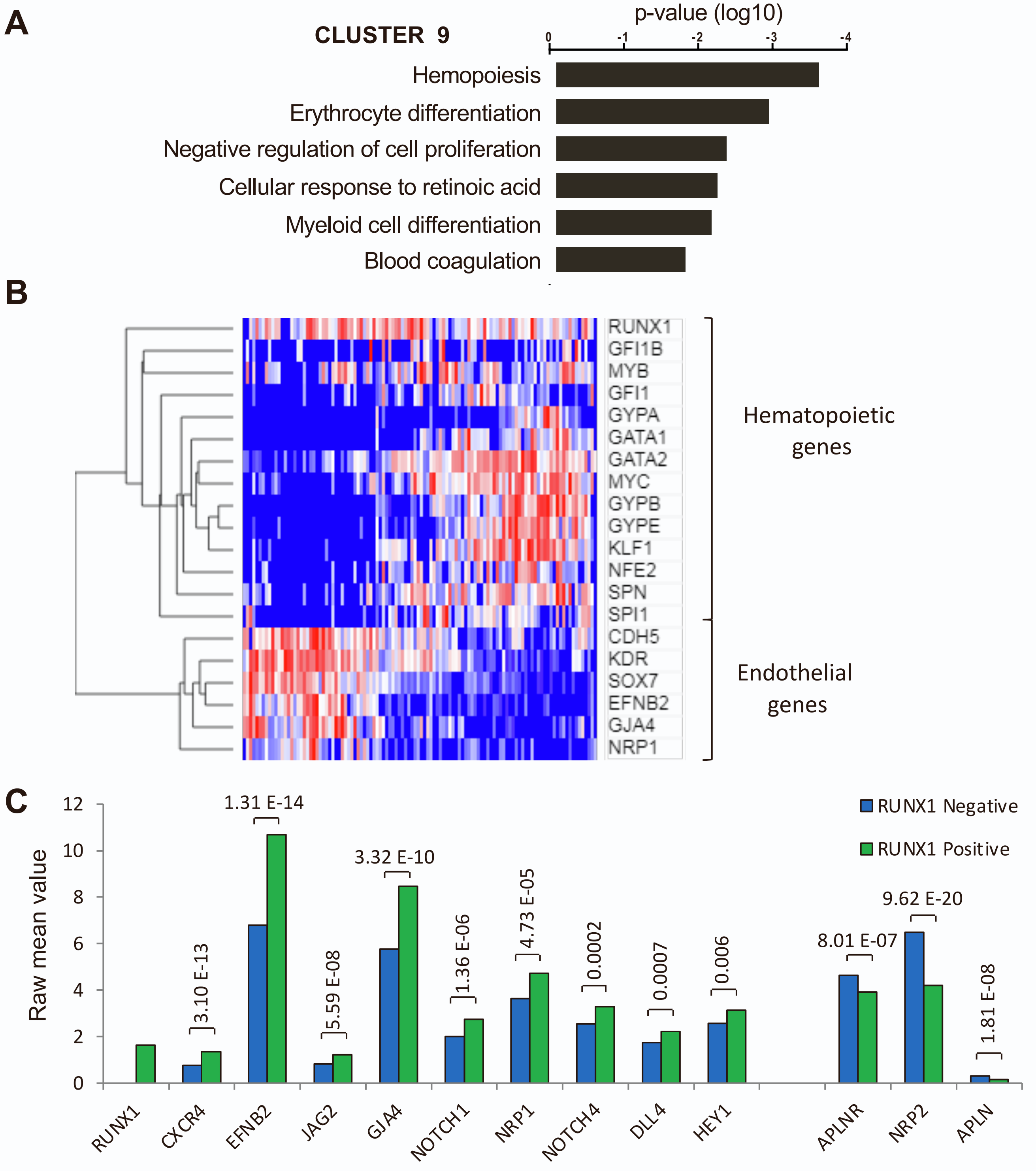

Figure S5: Characterisation of cluster 9. Related to Figure 4.
(A) GO terms enriched in Cluster 9 upregulated genes. (B) Heatmap of unsupervised hierarchical clustering of selected endothelial and hematopoietic genes expressed in cells of cluster 9. (C) Average expression levels from scRNA-seq dataset in RUNX1-positive (green bars) and RUNX1-negative (blue bars) cells within all endothelial clusters for selected arterial and venous genes. *P*-value are indicated on the graph.

Figure S5

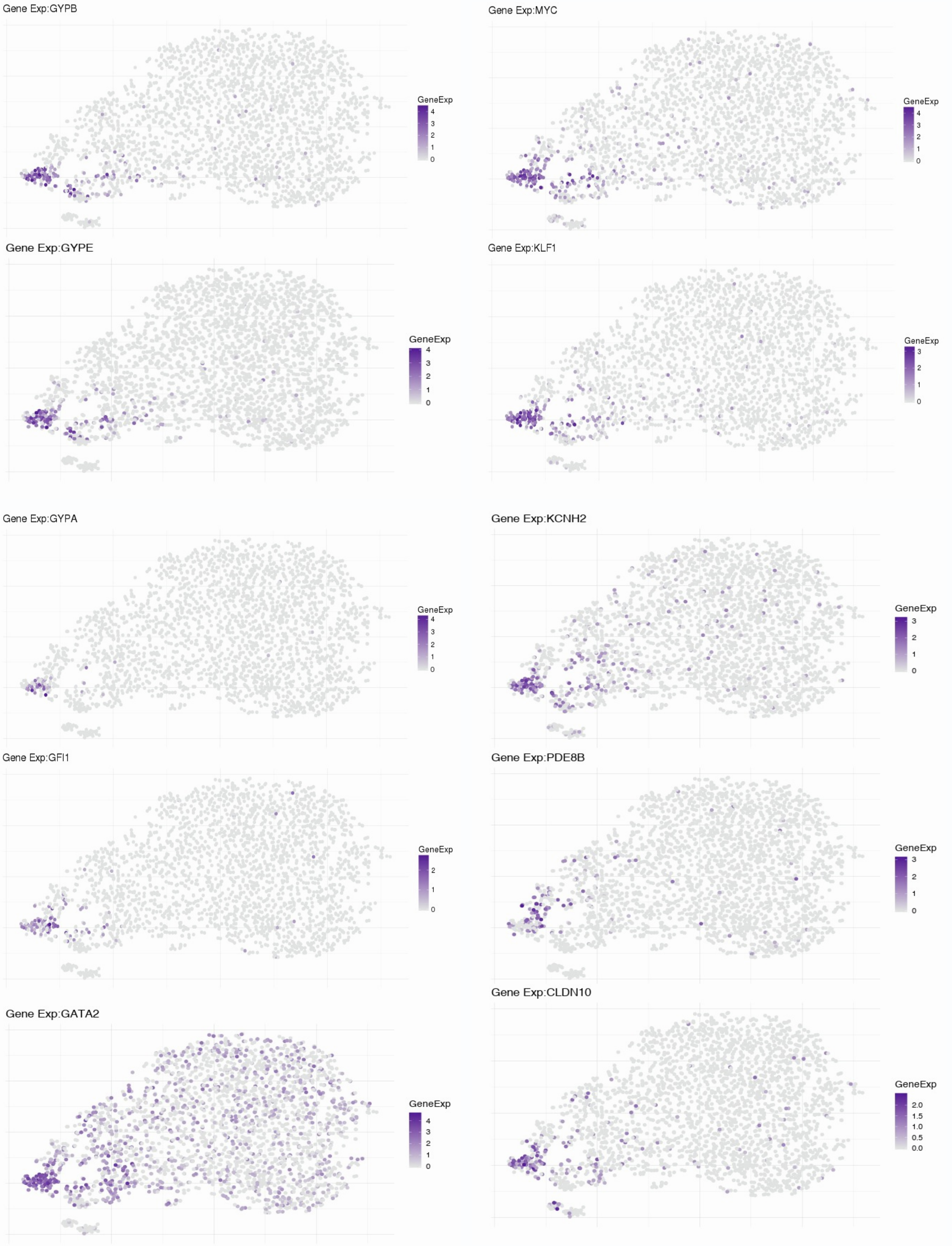

Figure S6: Gene expression across clusters. Related to Figure 5.
t-SNE plots showing the expression of genes which expression is significantly upregulated in cluster 9 compared to all other clusters.

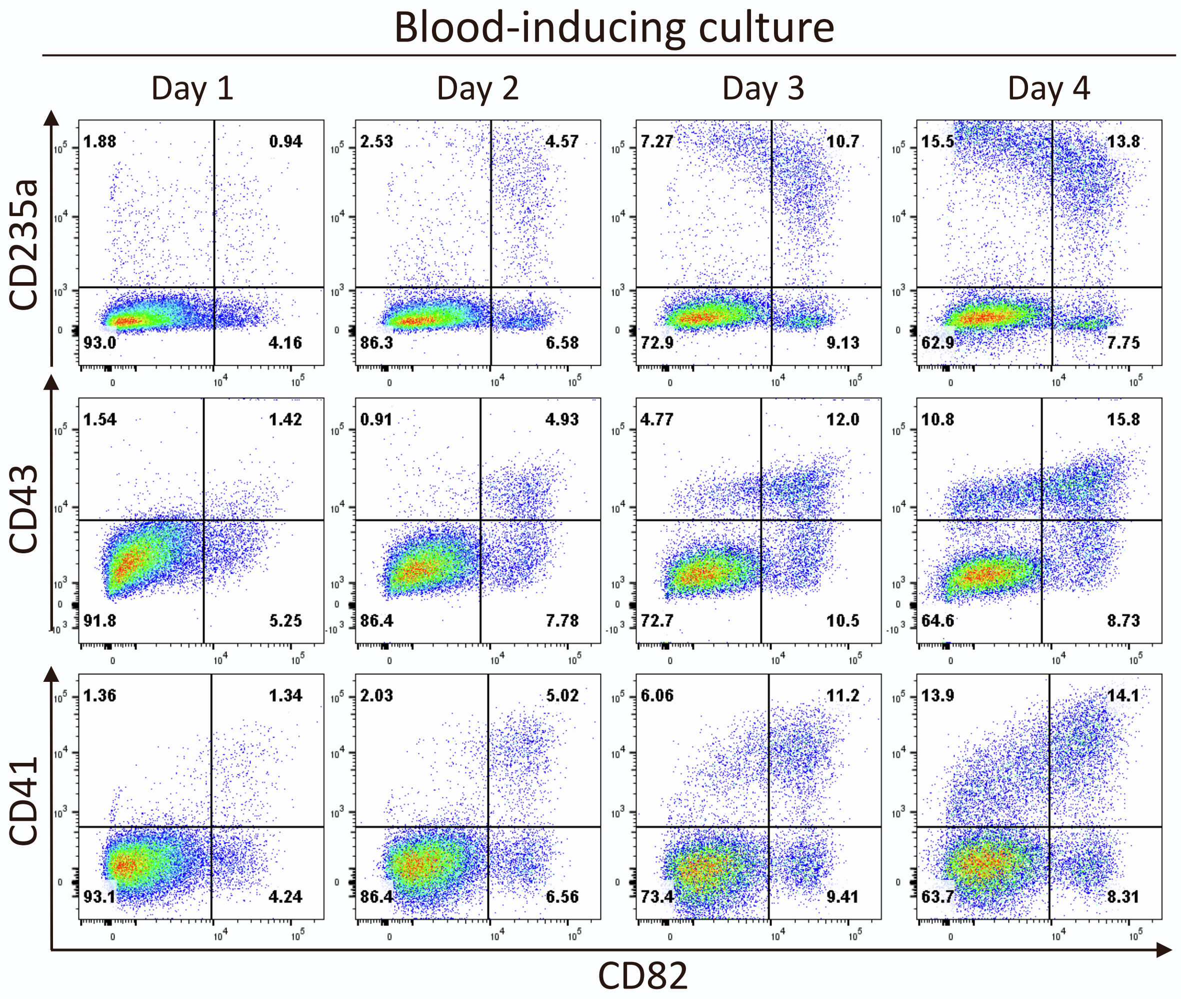

Figure S7: Dynamic of CD82 expression during blood cell emergence from differentiated H1 hESCs. Related to Figure 6.

Representative flow cytometry plots for the indicated cell surface markers at day 1, 2, 3 and 4 of blood cell emergence from $CD31^{+}CD144^{+}CD43^{-}$ cells isolated from day 6 of EB differentiation of H1 hESCs and cultured in hematopoietic inducing condition. Flow cytometry data are representative of two independent experiments.

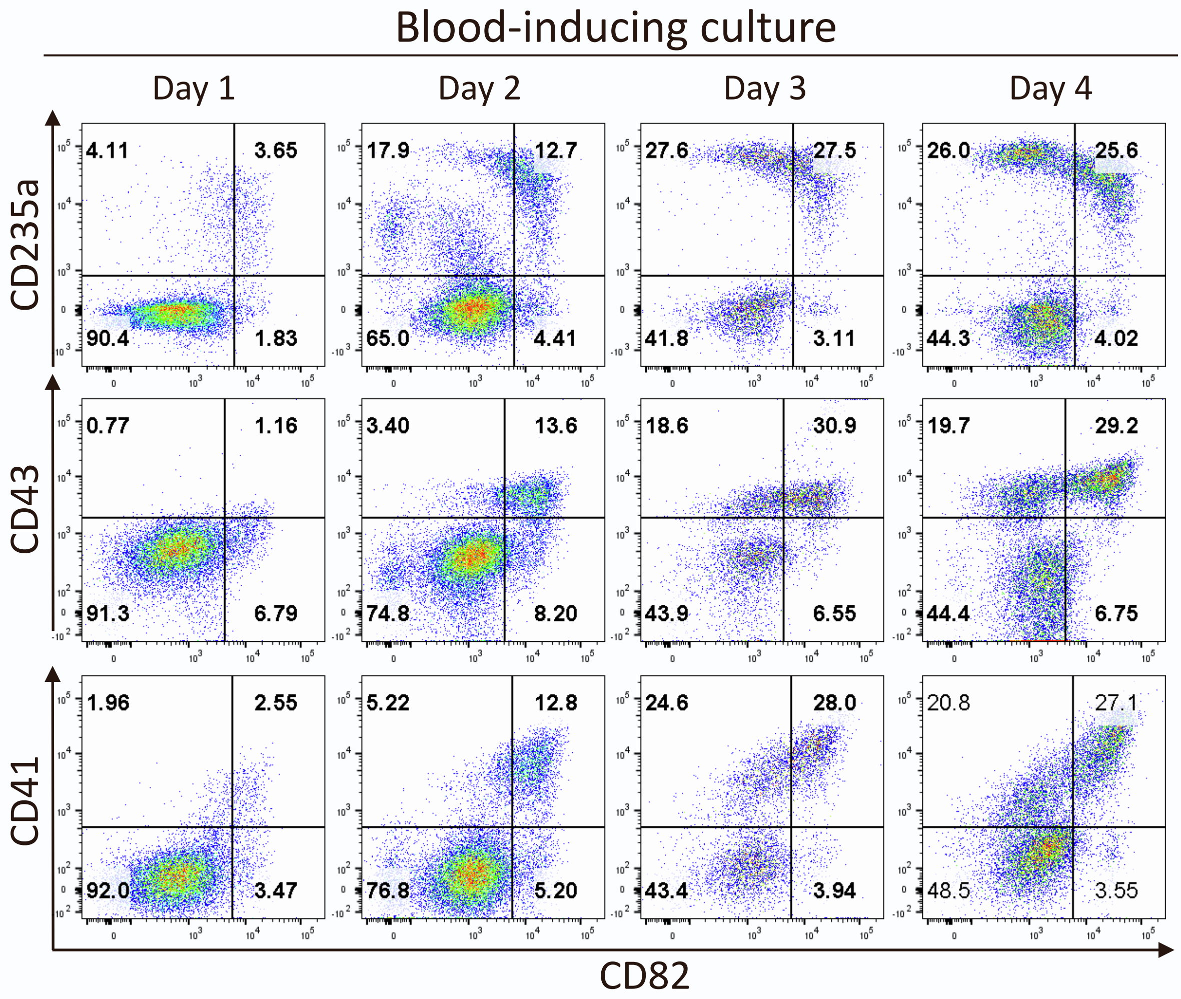

Figure S8: Dynamic of CD82 expression during blood cell emergence from differentiated GF4 iPSCs. Related to Figure 6.

Representative flow cytometry plots for the indicated cell surface markers at day 1, 2, 3 and 4 of blood cell emergence from CD31$^+$CD144$^+$CD43$^-$ cells isolated from day 6 of EB differentiation of GF4 iPSCs and cultured in hematopoietic inducing condition. Flow cytometry data are representative of two independent experiments.

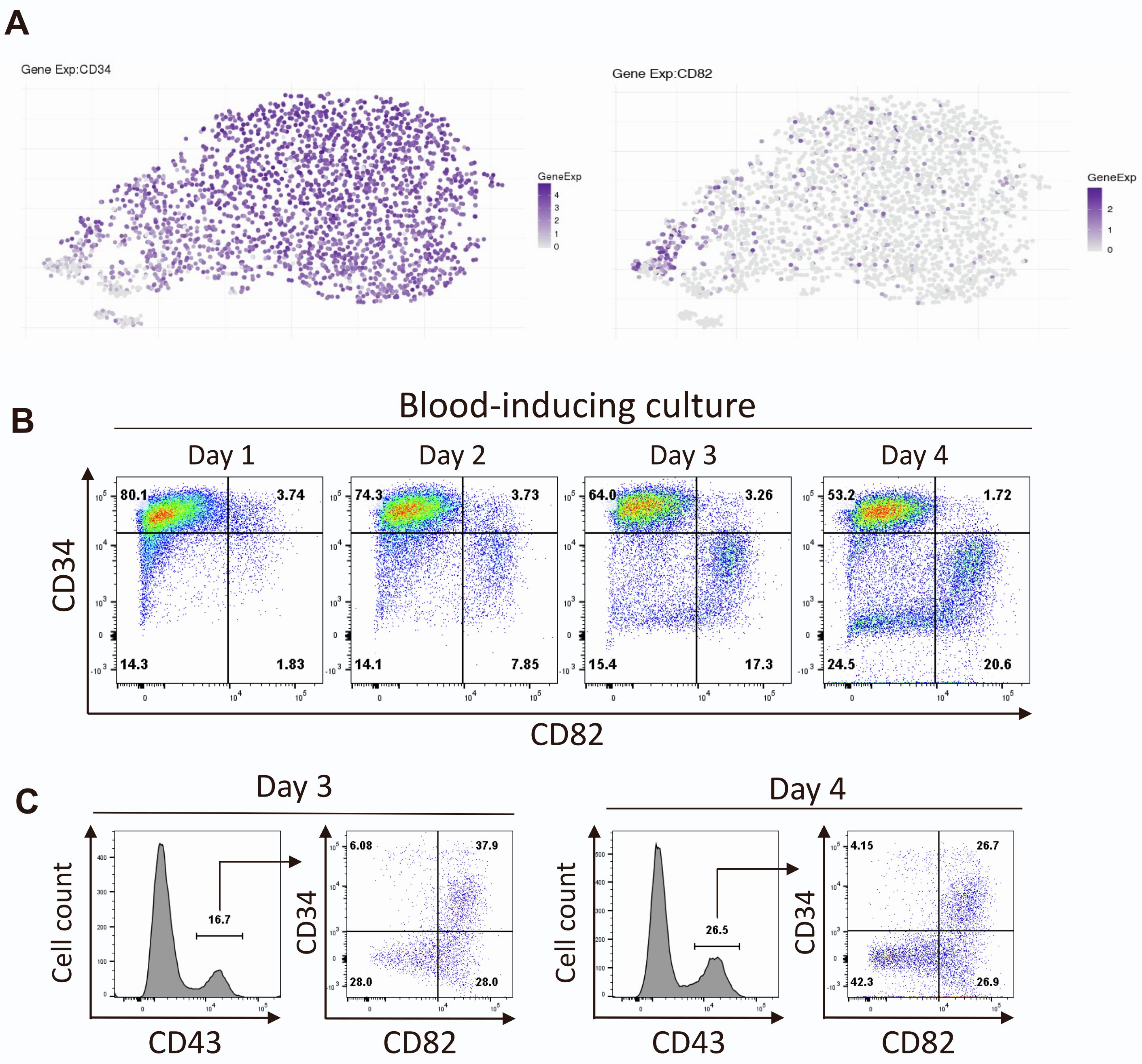

Figure S9: Co-analysis of CD82 and CD34 expression. Related to Figure 6.
t-SNE plots showing *CD34* and *CD82* expression across all clusters. (B) Representative flow cytometry plots for CD34 and CD82 at day 1, 2, 3 and 4 of blood cell emergence from $CD31^{+}CD144^{+}CD43^{-}$ cells isolated from day 6 of EB differentiation of *RUNX1b::VENUS* hESCs and cultured in hematopoietic inducing condition. (C) Representative flow cytometry plots of CD82 versus CD34 expression in $CD43^{+}$ cells at day 3 and day 4 of blood cell emergence. All flow cytometry data are representative of three independent experiments.

Supplemental table 1: Genes most down-regulated in cluster 9 relative to all other clusters. Related to Figure 5.

Gene name	LogFC	FDR
SOX17	-3.412778198	5.78E-47
RAMP2	-3.215549888	3.20E-97
ADGRL4	-2.743520292	6.77E-59
ARHGAP29	-2.73161832	5.32E-87
ID1	-2.664547243	1.21E-50
ID3	-2.574305153	1.01E-80
WWTR1	-2.538810345	5.96E-45
LDB2	-2.229648743	1.12E-48
HOPX	-2.139606926	2.23E-46
SMAGP	-2.122669477	4.58E-51
GSN	-2.093942051	2.14E-47
IGFBP4	-2.077466628	2.69E-82
IFI16	-2.05363072	4.51E-66
THY1	-2.048444396	1.40E-58
PLVAP	-1.760119338	1.04E-54
AP1S2	-1.69268576	5.76E-69
VIM	-1.678153348	1.09E-62
MARCKS	-1.603020522	1.68E-64
TMSB10	-1.522645529	1.24E-110
FSCN1	-1.452906133	1.98E-59
RDX	-1.43984293	5.64E-49
KDR	-1.385729208	3.82E-42
TMSB4X	-1.382804888	8.86E-56
SPTBN1	-1.349716996	1.02E-42
CALM1	-1.178234248	2.29E-46
MYL6	-0.922635948	1.05E-42
